# Malaria

**Published:** 1920-03

**Authors:** D. S. Davies, S. Kerfoot, Cecil Clarke


### MALARIA.

A discussion at a meeting of the Bristol Medico-Chirurgical Society on December 10th, 1919.

Dr. D. S. Davies opened the discussion with a short statement of the Public Health (Pneumonia, Malaria, Dysentery, etc.,) Regulations, 1919, issued by the Ministry of Health, which provide that malaria is a notifiable disease (unless previously notified within six months), and place upon the Medical Officer of Health definite duties of investigation, precaution and supervision, so far as concerns observance by the patient of prescribed measures of treatment and necessary measures to prevent the spread of infection; also where rendered expedient by the occurrence of indigenous cases, of taking measures, with the assistance of the Ministry, for the destruction of mosquitos.

Dr. Davies next dealt with the distribution of malaria cases introduced into the United Kingdom, quoting Lieut.-Colonel S. P. James,[1] who in a recent communication to the Medical Society of London points out that since March and up to November 24th nearly 14,000 cases of malaria had been notified, and the rate of notification was being maintained. Cases of malaria are now distributed almost everywhere in England and Wales. From the point of view of treatment the most important cases are those in

[1] *Brit. M. J.*, Dec. 6th, 1919, p. 743.

which the treatment of an acute relapse has not been successful in preventing a fatal issue. The deaths ascribed to malaria numbered 86 in 1917 and 108 in 1918, and if the deaths are maintained will this year number 280. Unfortunately, very many of the deaths occur in young men in the prime of life. Colonel James points out that there was evidence that in at least some instances death might have been prevented if all medical practitioners in England and Wales had a sufficient knowledge of diagnosis and treatment of malaria. *The Suggestions for the Care of Malaria Patients*, prepared by Colonel Sir Ronald Ross and Lieut.-Colonel S. P. James, and issued by H.M. Stationery Office, deal with treatment and prevention in detail.

Dealing with the disease as it has affected Bristol :—

110 cases have been reported.

109 of these cases are ex-service men.

1 case, a woman, who contracted the disease some years ago when engaged in missionary work in Ceylon and Singapore.

3 cases have proved fatal.

Of the total cases notified 102 were reported by private medical practitioners since March, 1919, and 8 cases, pensioners, were reported by the Ministry of Health since November, 1919, who received information from the Local Medical Board.

Three fatal cases occurred : one contracted in Palestine in a man of 48, demobilised in March, 1919, died June, 1919 ; the second in a man of 43, a naval reservist stationed in Sierra Leone two years, invalided in September, 1916, under treatment at a local hospital, died June, 1919 ; the third a man of 21, from Salonika, died December, 1919.

*Indigenous Cases.*—The total number of cases of malaria contracted in England which have been discovered and notified since 1917 amounts to 414 (up to Nov. 24th, 1919).

Among these cases occurring in the civil population the great majority (75 per cent.) have been in children under 15. Some of the patients received quite inadequate quinine treatment, even after a definite diagnosis of malaria had been made. No indigenous cases have been reported in Bristol.

Although all demobilised soldiers suffering from malaria are entitled to receive treatment from an insurance practitioner, many who still suffer have not applied for treatment since they were demobilised. Consequently, the Ministry of Pensions have recently arranged to forward particulars of such cases to the Ministry of Health, who distribute the names to the Medical Officers of Health of the districts concerned. The number of reports now received by the Ministry averages 300 a week. Some of these neglected cases have communicated the disease to others, and it is obvious that prevention of the spread of malaria in this country is intimately bound up with arrangements for ensuring the satisfactory treatment of demobilised soldiers.

In 1917 and 1918 most of the sixty-seven indigenous cases of malaria occurred in North-East Kent, near the mouth of the Thames, and in general it is those districts formerly known to be malarious or which favour the presence and multiplication of the Anopheles mosquito in which danger is most to be feared. Three species of Anophelines are found in the British Isles: (1) A. Maculipennis, (2) A. Bifurcatus, (3) A. Plumbeus (nigripes), a comparatively rare species, but not uncommon in some districts; and of these the first two are known to be capable of transmitting the parasite of malaria. A. Maculipennis, to be recognised by its unbanded legs and palpi and its four spots in the field of the wing, but no spots on the upper margin, appears to be more common than A. Bifurcatus, possibly because as it frequents houses it comes more under observation. It has been observed that Anophelines, though much commoner in the Fen districts and marshy ground as at New Romney, may be found anywhere where suitable conditions for the larvæ exist. Such conditions are generally not met with in the more densely-populated portions of large towns, but rather in the outskirts.

In order to deal with the threat of malaria recrudescence, especially in those districts in which the occurrence of indigenous cases has pointed to the effective presence of the malaria-transmitting mosquito, the Ministry of Pensions has recently instituted a malaria clinic in London and other places, through which the systematic examination and treatment of pensioned soldiers may be secured. Locally pensioners come within the purview of the War Pensions Committee and their medical referees, and some hospital accommodation is available for suitable cases, but no clinic has yet been established. The comparative paucity of effective mosquitoes in the district may promise immunity, but the records of Bristol shows how widely the home-coming reservoirs of potential infection are distributed in the city, and presumably also in the more dangerous rural surrounding areas, and this alone warrants an attitude of precaution. The chief danger is that a man whose disability is assessed by the Medical Pensions Board at *nil* retires into civil life and may neglect to apply to the local War Pensions Committee in the event of a relapse, as he should do in order to be examined by the Medical Referee. It behoves the Medical Officer of Health to maintain some surveillance over such men when they cease to be pensioners, and this is secured as far as possible.

Dr. S. KERFOOT, after giving a general survey of the various clinical manifestations of the disease as hitherto described, related some of his own interesting experiences amongst malaria patients in the Near East. His remarks were as follows:—

I have seen catalepsy mistaken for malarial coma. I remember a patient who was sent to me from another hospital for treatment with galyl, after having had intravenous injections of quinine for supposed malarial coma, who on the first day of admission again became comatose. His previous hospital report showed that the malarial parasite had never been found, although repeated examinations of his blood were made, neither did his temperature chart show any definite attacks of fever. On admission in the morning, which was Sunday, I gave him an intramuscular injection of quinine as routine treatment; but in the evening he was found by the sister of the ward to be comatose, and as I was away a colleague gave him an intravenous injection of quinine, when he regained consciousness, but the next morning he again became comatose. Such an unusual procedure put me on my guard, and on careful examination I found that he was in a cataleptic state. Hypnotic suggestion by a colleague and a firm hand procured a cure, and although he remained in hospital for another two months, and not one grain of quinine was administered during that time, yet he never again lapsed into his old ways or showed symptoms of malaria, nor was a parasite ever found in his blood.

Proceeding to the treatment of malaria, quinine when properly administered must be looked upon as a specific, and as a general rule any fever simulating malaria which resists quinine properly administered is not malaria, and some other disease must be looked for. An initial dose of calomel should always be given in the commencement of treatment of malaria, and aspirin relieves the headaches and the pains in the back and limbs, and promotes the sweating stage, and sponging and baths should be resorted to for high temperature. Quinine when administered by the mouth should not be given in the form of tablets but in solution, and the bihydrochloride of quinine is much better borne by the stomach than the sulphate, and is for some reason more efficient, although more expensive, and it is readily soluble in water. Quinine administered for prophylaxis is entirely erroneous because it is not adequate treatment and keeps the malarial attacks in abeyance, killing most of the asexual forms of the parasite and rendering the sexual forms latent, so that the disease is not discovered until well established and the parasites immunised to quinine.

Now in the *benign tertian* form of malaria 15 grains of quinine three times a day should be administered by the mouth for one week, and 10 grains three times a day for two more weeks. This is generally sufficient, but in some resistant cases and in cases which show a one-day relapse every eight or ten days one or two intramuscular injections of 15 grains of quinine should be added to the treatment. Iron and arsenic should be administered in the convalescent stage.

In *quartan* malaria, which is much less common, the same method should be carried out as for the benign tertian. This form is more difficult to cure, but the attacks are not so severe.

In *malignant* malaria quinine by the mouth is totally inadequate, and 15 grains of the bihydrochloride of quinine intramuscularly should be given twice a day for one week, with a midday dose of 15 grains by the mouth, and 10 grains three times a day by the mouth for three more weeks, with one weekly injection of 15 grains intramuscularly. If any relapse occurs the same treatment should be repeated.

It is particularly in malignant malaria that we see pernicious symptoms, and for the more severe of these it is necessary to give quinine intravenously. Twenty grains of the bihydrochloride should be given in about half a pint to a pint of sterile normal saline at body temperature twice a day for one, two or three days according to the severity of the attack, and then followed intramuscularly and by the mouth as in an ordinary malignant attack. In comatose cases I have known as much as 200 grains given in two days in three daily intravenous injections with excellent results and with no toxic symptoms to speak of. I believe I am correct in stating that no case of quinine amblyopia has occurred with a less dose than 120 grains. At least that was so in the Salonika area.

Then we meet the chronic relapsing cases with marked cachexia. These cases sometimes respond to quinine intramuscularly or intravenously, but it is for these cases, for those with profound anæmia and those with quinine idiosyncrasy that I would specially advise intravenous injections of galyl. Three injections (0.3 gm.) once a week for three weeks with quinine treatment in between (either by the mouth or intra-muscularly as considered necessary) is excellent treatment. The effect of the galyl on the blood corpuscles is most marked, and no other treatment would give like results in this respect, which is very important in a disease which is characterised by extreme destruction of the R.B.C. The patients in a few days take on a markedly healthier appearance. The sallow skin becomes bright and tinged with colour, and the patient feels immensely better. I will mention a few cases in which galyl was given. They had two weekly injections, and the second R.B.C. count was taken on the eighteenth day.

No. I.—Before the galyl: 1,290,000 reds with 20 per cent. hæmoglobin.
In eighteen days: 4,110,000 reds with 70 per cent. hæmoglobin.

No. II.—Before the galyl: 2,060,000 reds with 50 per cent. hæmoglobin.
In eighteen days: 4,370,000 reds with 70 per cent. hæmoglobin.

No. III.—Before the galyl: 2,170,000 reds with 55 per cent. hæmoglobin.
In eighteen days: 3,510,000 reds with 70 per cent. hæmoglobin.

Then the effect of galyl upon the parasite is also marked. The temperature falls almost immediately and the attack is terminated, whilst the sexual forms of the parasite, which probably were absent or few in the circulating blood, are turned out from the internal organs where they have been lying latent into the circulating blood in the same way as the trypanosome is turned out by atoxyl, and perhaps are induced to fertilise and split up by parthenogenesis into asexual forms, which are more readily killed by treatment, and then the parasites gradually disappear. I have given as many as eleven injections of 0.2 gm. to a man who had quinine idiosyncrasy and was almost moribund, and for whom galyl alone had to be depended upon as treatment, with excellent results. Salvarsan, kharsivan, phosphorated oil and sodium cacodylate, which is an unstable arsenical preparation, as injections, and other drugs by the mouth, such as methylene blue and other forms of arsenic have been tried with poor results.

Then we come to the transfusion of whole blood as treatment in serious cases where the hæmolysis is extreme and the R.B.C. count is down to 1,000,000 or even less, and many cases owed their lives to this treatment, as when the R.B.C. count has fallen so low the resistance to the parasite seems to have disappeared. Indirect transfusion must be used, as direct transfusion would mean infection for the donor. The blood of suitable donors must be tested with the blood serum of the recipient for agglutination, since it has been found that only one out of three is suitable.

A spherical glass vessel is used of about two pints capacity with a vein cannula below and a tubed cork above. This is sterilised and coated on the inside with sterile paraffin wax to prevent coagulation of the blood. The cannula is inserted into the distal end of the slit vein of the donor by one surgeon and the blood allowed to rise by its own pressure into the vessel, which, when sufficiently filled—say about a pint and a half—is withdrawn and passed to another surgeon, who allows the blood to flow into the central end of the recipient's slit vein, helped by the pressure from an air-pump which is attached to the tube in the cork at the top of the vessel. A blood count the next day will show a rise of 500,000 to 1,000,000 reds, which gradually falls for two or three days, then remaining stationary, and finally the patient's own physiological efforts continue the increase.

Dr. Cecil Clarke contributed the following remarks :—

There seems to be general agreement that the difficulties encountered in treating malaria acquired in Macedonia were greater than those met with in the case of malaria acquired in Mesopotamia. As a result of extensive trials, Sir Ronald Ross reported that no matter how big the dose of quinine or what route was chosen for its administration frequent relapses occurred, about 30 per cent.

An opportunity of comparing malaria acquired in East Africa with that of Macedonia presented itself in the early months of 1918. Units from both areas of warfare had arrived in France, and were to be examined both generally and by blood film examination as to their fitness for front line work and freedom from malarial infection. No parasites were found in the men from East Africa (twenty films), whereas 40 per cent. of the men who had served in Macedonia showed parasites, benign tertian in type, in the sixty films examined. Despite continuous treatment with quinine, anæmia and enlargement of the spleen were present in varying degrees. The mortality rate for 1916 (January to November) was 1.0 per cent. In 114 fatal cases investigated 65 died within two days of admission to hospital.

Two observations in connection with the morbid anatomy of malignant malaria are worthy of mention.

1. The association of *acute cardiac failure or syncopal attacks* with fatty degeneration of the heart muscle. Of forty-five such tissues examined by Dudgeon and Clarke twenty-three showed a varying degree of fatty degeneration, well marked in eight and less so in fifteen. The microscopical appearances are indistinguishable from the fatty change seen in fatal diphtheria toxæmia. If it occurs in fatal pernicious malaria there is every reason to presume that it is present in the case that recovers. Even if limited or slight in a microscopical section it must represent a considerable fraction of the heart muscle. Its bearing on the immediate treatment of a relapse of fever is obvious.

2. The occurrence of hemorrhages into the lungs. The hemorrhages are usually limited in character but fairly wide in distribution ; exceptionally massive consolidation of lung tissue results. These changes were present in twenty of thirty-three tissues microscopically examined. True lobular pneumonia was present in five cases and lobar pneumonia in one case ; the hemorrhages afford a suggestive anatomical basis for a " malarial pneumonia."

Quartan infection was so extremely rare as to be negligible. Both in 1916 and 1917 primary infections of benign tertian were seen first in the last week of April and first week of May. Primary malignant tertian infections were not met with before the early days of June. The point was easy of determination in both years. In 1916 the majority of the troops had arrived in the latter months of 1915, and in 1917

there were many drafts who had not spent the previous summer in Macedonia. Willoughby and Cassidy state that at one camp where the predominant Anophelines were A. Pseudopictus and A. Superpictus the incidence of subtertian infection was very heavy. The other known carriers in the country were A. Maculipennis and A. Bifurcatus. Wenyon found that the carrier most commonly met with was A. Maculipennis, next in order of frequency came A. Superpictus.

Although it must be admitted that the administration of prophylactic quinine given with a view to preventing the development of malaria failed as a whole in Macedonia, it is possible to bring forward figures to show that the administration of quinine influenced favourably the amount of sickness. As regards the first point, Willoughby and Cassidy state: "From our experience we may say at once and emphatically that the prophylactic value of quinine in the doses given has been at all events so incomplete and questionable that none of the other precautions can for a moment be neglected in favour of such practice however universally it may be carried out." It may prevent the development of clinical malaria. A unit was stationed in July, 1916, in an area where the incidence of malaria was very high, and was moved in August to an area where primary cases of infection and mosquitoes were for the country infrequent. No case of malaria in the unit, varying from sixty to seventy in strength, was diagnosed microscopically until the months of January to March (the coldest months) when twenty-three cases occurred. Since arrival in the new area the unit had been taking 10 grs. daily in liquid form.

C. H. Treadgold[1] in a review of the whole question of

[1] C. H. Treadgold, "The Prophylactic Use of Quinine in Malaria," *Brit. M. J.*, May 11th, 1918.

prophylactic value of quinine in Macedonia considered that the general course of the disease may be adversely influenced by the previous taking of quinine.

The behaviour of the two parasites in the culture tube is distinctly different. Using what proved to be a method unfavourable in comparison with the original technique of Bass, Colonel Dudgeon and myself obtained a positive result in fourteen out of eighteen strains of the malignant parasite examined and a completely negative result with the benign tertian parasite in twelve cases. The serum employed was that of the patient, and, as Dr. Nierenstein has determined, the concentration of quinine in the serum attains a strength no greater than 0.8 gramme per litre whatever the dose given. The patients were with two exceptions being treated for malaria. The negative result in the benign tertian infections is perhaps to be assigned to the concentration of quinine in the culture tube. Repeating Bass's original technique, we found that the benign tertian parasite again failed to grow if the serum had been derived from a patient who had received more than 20 grains in the preceding twenty-four hours. This is in direct contrast to the behaviour of the malignant parasite. A strain derived from a patient who had been taking quinine daily for five months in doses of 10 grains and 40 grains in the preceding twenty-four hours developed typically and was followed for thirty-eight hours.

It is well known that the benign tertian parasite usually disappears from the peripheral blood in the twenty-four hours following a full dose of quinine. The effect of quinine on the sexual forms of this parasite was not so certain. C. H. Treadgold examined in the early months of 1917 125 men taking either quinine in doses of 30 grains daily to 15 to 30 grains bi-weekly or in twenty-six cases no quinine except during relapses. Sixty-five showed parasites in the films and 90 per cent. gametes. Quinine has no ascertainable certain effect on the sexual forms of the malignant parasite. Its effect on the asexual forms was not so certain or so rapid as in the case of the benign tertian infection.

The mode of action of quinine still remains unknown, but in the culture tube it can be easily ascertained that the leucocytes can engulf and destroy full-grown parasites. Moreover, it is usual to find a complete disappearance of parasites if left in contact with serum and leucocytes for thirty-six hours or more.

Bass has shown that without the presence of dextrose in the serum of the culture tube, the strength at least 0.5 per cent., no development takes place. As is well known, the amount of dextrose in the circulating blood is practically a constant, 0.08 per cent. to 0.1 per cent. It has been shown that under conditions which cause fatigue the blood sugar may rise to a greater height than 0.1 per cent., and takes three to four hours to fall to its original level. The circulating dextrose is constantly being taken up and used by the muscles. Clinically, relapse is prone to occur after a long, tiring day, a spell of cold weather, in fact, anything which means extra muscular work. The life of the infantry in Macedonia provided many such opportunities, and relapse in a man recently returned from a convalescent camp was frequent.

It may be that the increased dextrose content of the blood, consequent on hard muscular exertion, plays some part in the pathology of a clinical relapse of malaria.

